# CANCER: Childhood Leukemia and Proximity to Nuclear Power Plants

**Published:** 2009-10

**Authors:** Adrian Burton

**Affiliations:** **Adrian Burton **is a biologist living in Spain who also writes regularly for *The Lancet Oncology*, *The Lancet Neurology*, and *Frontiers in Ecology and the Environment*

Since the first report of increased childhood leukemia rates around Britain’s Sellafield nuclear power plant (NPP) in 1983, controversy has surrounded the possible link between the disease and proximity to nuclear reactors. Twenty‐five years later the debate rages on, with different studies yielding seemingly contradictory findings. A public sensitized to the dangers of nuclear power might well ask the question: why aren’t we sure by now?

“The many studies that have been performed are difficult to compare because of differences in their methodology,” explains John Bithell, honorary visiting fellow at the Childhood Cancer Research Group, University of Oxford. These differences include the age groups studied, the geographical areas considered, and potential confounding factors such as socioeconomic status.

“Moreover,” says Bithell, “we are looking at a very small effect in terms of the actual numbers of sick children involved, and the statistical tools used have not always had the necessary power to allow conclusions to be drawn. Add all this to the fact that we do not actually know [all] the causes of leukemia, and you can see that it becomes difficult to firmly establish a link between it and NPPs.”

According to industry records and the presently accepted canon of radiobiology as defined by national and international radiation regulatory bodies, children living near NPPs are exposed to doses of radiation orders of magnitude below those thought to cause leukemia. However, Rudi Nussbaum, a professor emeritus of physics and environmental sciences at Portland State University, says evidence of extreme radiation sensitivity of embryos and fetuses has been largely ignored in this canon, as have reports of low‐dose health effects from inhaled or ingested radioactive fallout at large distances from the Chernobyl nuclear disaster.

It is challenging—but not impossible—to estimate the effective dose of ionizing radiation from an NPP to which a child may have been exposed over the years, says Joseph Mangano, executive director of the nonprofit Radiation and Public Health Project. Measurements of in‐body levels of radioactivity are critical to resolve this issue, he says. Perhaps the most feasible way to take such measurements is to test for bone‐seeking isotopes in baby teeth.

The German KiKK (Kinderkrebs in der Umgebung von Kernkraftwerken) study, a case–control study described by Peter Kaatsch and colleagues in the 15 February 2008 issue of the *International Journal of Cancer*, found that children under age 5 years living within 5 km of an NPP were at more than double the normal risk of developing leukemia. Although similar links have been reported by several other authors, a number of ecological studies suggest that children living near NPPs are at no greater risk than other children. The KiKK study pinpointed the distance of individual case homes from each of the 16 German NPPs and was therefore better able to classify exposure than ecological studies, which use approximate distances to classify exposure. Nussbaum argues that ecological studies tend to average out the significant risk–distance association, especially when the number of cases is small.

Alfred Körblein, a retired physicist formerly with the Munich Environmental Institute, a German nongovernmental organization, makes a similar observation regarding a recent re‐analysis of the KiKK data published in volume 105, issue 42 (2008) of *Deutsches Ärtzeblatt International*, which used approximate distances to estimate exposures. “This ecological analysis of the same data yielded only a nonsignificant 41% increase [in leukemia incidence] in the 0‐ to 5‐km zone compared to the 119% increase in the superior case–control analysis,” he says. “But that’s what you’d expect when using the weaker ecological approach.”

In fact, in their 2008 ecological analysis Kaatsch and colleagues wrote, “Since the determination of distance using the central point of the community was much less exact than using individual residential addresses, as in the case–control study, a correspondingly less clear measure of effect was to be expected. In this respect the two approaches are not contradictory.” Co‐author Claudia Spix, deputy director of the German Childhood Cancer Registry at the University of Mainz, explains, “We wished to demonstrate the basic agreement of the results obtained by both approaches.”

Some researchers conclude that the consistency between the KiKK study findings and comparable ecological studies proves the real controversy is no longer about the validity of the leukemia–distance association. “Rather,” says Nussbaum, “it involves both the mechanism of the disease initiation and the public health implications of the confirmed leukemia clusters near NPPs.”

Others believe this stance is premature, given that the KiKK researchers were unable to adjust for any potential confounders besides sex and age—leaving the possibility that some factor besides radiation caused the children’s disease. Currently, however, ionizing radiation is the only established environmental risk factor for childhood leukemia, according to a review by Martin Belson and colleagues published in the January 2007 issue of *EHP*. At the very least, Nussbaum argued in the July–September 2009 issue of the *International Journal of Occupational and Environmental Health*, the KiKK study points out the need for a critical reexamination of the fundamental assumptions and models underlying current radiation safety standards and regulations.

In a time when many governments are exploring alternatives to fossil fuel–based energy, nuclear power also remains controversial because of unresolved questions about the safe storage of radioactive waste and the potential for radioactive contamination stemming from accidents or terrorist attacks. In any area where science, politics, and powerful commercial interests meet, it is critical to focus on the science, says Mangano. “Studies of childhood cancer and leukemia from exposure to nuclear reactor emissions have been clouded by political factors,” he says. “A challenge to objective, dispassionate science is to overcome this and help policy makers make the right decisions in this emotive area.”

## Figures and Tables

**Figure:**
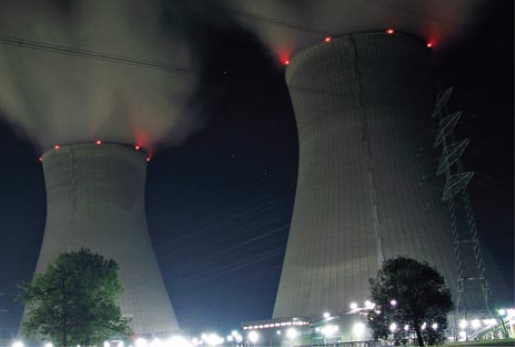
Gundremmingen Nuclear Power Plant Günzburg, Bavaria
